# Letter from the Editor in Chief

**DOI:** 10.19102/icrm.2022.130405

**Published:** 2022-04-15

**Authors:** Moussa Mansour


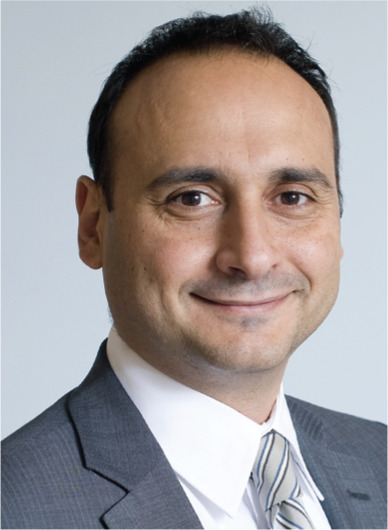


Dear readers,

The field of atrial fibrillation was well represented during the recent annual scientific meeting of the American College of Cardiology. Of particular note, a late-breaking clinical trial assessed peri-device leak after left atrial appendage occlusion (LAAO) in 51,333 patients enrolled in the National Cardiovascular Data Registry LAAO Registry.^1^ According to the study authors, 37,696 (73.4%) patients had no leak, 13,258 (25.8%) had small leaks, and 379 (0.7%) had large leaks. Interestingly, patients with a small leak had higher rates of thromboembolic and bleeding events compared to those with no leak.

This work corroborates the findings of a similar study presented by Reddy et al. during the American Heart Association scientific meeting in November 2021,^2^ which enrolled patients who received the WATCHMAN™ device (Boston Scientific, Marlborough, MA, USA) in the PROTECT-AF and PREVAIL studies. Among 1,053 patients, leaks were found in 40% at 45 days (via transesophageal echocardiography) and 28% at 12 months. Compared to patients with no leak, those with a peri-device leak had a greater rate of non-disabling stroke but similar rates of death and disabling stroke.

Some earlier studies did not find a correlation between peri-device leak and thromboembolic events, but they enrolled limited numbers of patients, and I believe that any doubt about such a correlation will be put to rest given the recent findings. Now, how will this recent knowledge affect clinical practice? It must be remembered that the rate of thromboembolic events is very small in this subgroup of patients, as demonstrated in the 2 studies described above. Moreover, when a stroke occurs after WATCHMAN™ implantation, it is often mild and rarely disabling. Third, the vast majority (if not all) of participants in these 2 studies received first-generation WATCHMAN™ devices. The PINNACLE study demonstrated that the newer-generation WATCHMAN™ FLX device is superior to the first-generation device and associated with a significantly lower rate of peri-device leak. As a result, it is unlikely that peri-device leaks will be a significant clinical problem going forward. The decision for closing a peri-device leak will continue to be challenging, and the safety and efficacy of the procedure for closing such leaks have not been proven. I believe it would be prudent to proceed with a personalized approach that considers the comorbidities and particular anatomy for every patient.

I hope that you enjoy reading this issue of *The Journal of Innovations in Cardiac Rhythm Management*, and best wishes for a productive annual scientific meeting of the Heart Rhythm Society.

Sincerely,


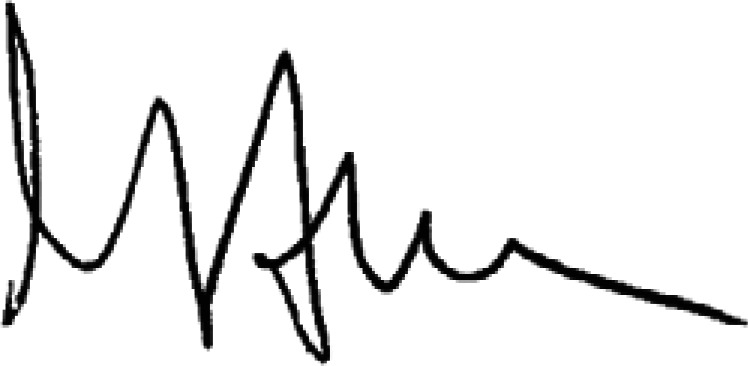


Moussa Mansour, md, fhrs, facc

Editor in Chief

*The Journal of Innovations in Cardiac Rhythm Management*

MMansour@InnovationsInCRM.com

Director, Atrial Fibrillation Program

Jeremy Ruskin and Dan Starks Endowed Chair in Cardiology

Massachusetts General Hospital

Boston, MA 02114
